# Quantitative Trait Loci Mapping of Adult Plant and Seedling Resistance to Stripe Rust (*Puccinia striiformis* Westend.) in a Multiparent Advanced Generation Intercross Wheat Population

**DOI:** 10.3389/fpls.2021.684671

**Published:** 2021-12-23

**Authors:** Sandra Rollar, Manuel Geyer, Lorenz Hartl, Volker Mohler, Frank Ordon, Albrecht Serfling

**Affiliations:** ^1^Julius Kühn Institute (JKI) – Federal Research Centre for Cultivated Plants, Institute for Resistance Research and Stress Tolerance, Quedlinburg, Germany; ^2^Bavarian State Research Center for Agriculture, Institute for Crop Science and Plant Breeding, Freising, Germany

**Keywords:** stripe rust, *Yr* genes, MAGIC population, simple interval mapping, QTL

## Abstract

Stripe rust caused by the biotrophic fungus *Puccinia striiformis* Westend. is one of the most important diseases of wheat worldwide, causing high yield and quality losses. Growing resistant cultivars is the most efficient way to control stripe rust, both economically and ecologically. Known resistance genes are already present in numerous cultivars worldwide. However, their effectiveness is limited to certain races within a rust population and the emergence of stripe rust races being virulent against common resistance genes forces the demand for new sources of resistance. Multiparent advanced generation intercross (MAGIC) populations have proven to be a powerful tool to carry out genetic studies on economically important traits. In this study, interval mapping was performed to map quantitative trait loci (QTL) for stripe rust resistance in the Bavarian MAGIC wheat population, comprising 394 F_6 : 8_ recombinant inbred lines (RILs). Phenotypic evaluation of the RILs was carried out for adult plant resistance in field trials at three locations across three years and for seedling resistance in a growth chamber. In total, 21 QTL for stripe rust resistance corresponding to 13 distinct chromosomal regions were detected, of which two may represent putatively new QTL located on wheat chromosomes 3D and 7D.

## Introduction

The biotrophic fungus *Puccinia striiformis* Westend. f. sp. *tritici* Eriks. is the causal agent of stripe rust and is one of the most important foliar diseases of wheat, which accounted for 25% of global cereal crop production in 2018 (Food and Agriculture Organization of the United Nations (FAO), 2020). Particularly prevalent in the temperate and maritime wheat growing regions, stripe rust can cause yield losses up to 70% mainly by reducing photosynthesis and taking assimilates from the host plant (Chen, 2005; Jagger et al., 2011; Rosewarne et al., 2012). In agricultural production systems, the application of fungicides, as well as the growing of resistant cultivars are currently used to control stripe rust, of which the latter is the most economically safe and environmentally friendly approach to avoid yield losses. To date, about 82 stripe rust resistance genes (*Yr* genes) have been unequivocally identified, but a lot more temporary designated genes and quantitative trait loci (QTL) have been reported and mapped across the whole wheat genome (McIntosh et al., 2019; Yang et al., 2019a). Of these, *Yr5, Yr7, Yr10, Yr15, Yr18, Yr36, Yr46*, and *YrSP* have already been cloned and characterized as intracellular nucleotide-binding leucine-rich-repeat receptors (*Yr5, Yr7*, and *YrSP*), putative kinase-pseudokinase protein (*Yr15*), transporters (*Yr18* and *Yr46*), or wheat kinase start 1 (*Yr36*) (Fu et al., 2009; Krattinger et al., 2009; Liu et al., 2014; Moore et al., 2015; Klymiuk et al., 2018; Marchal et al., 2018). In addition, resistance genes, such as *YrAS2388R* derived from *Aegilops tauschii* and YrU1 derived from *Triticum urartu* have recently been cloned, encoding a nucleotide oligomerization domain-like receptor (NLR) and a coiled-coil-NBS-leucine-rich repeat protein with N-terminal ankyrin-repeat and C-terminal WRKY domains, respectively (Zhang et al., 2019; Wang et al., 2020).

Mainly two different types of resistance are described based on criteria, such as inheritance, specificity, plant growth stage, and temperature (Chen, 2013; Liu et al., 2018). The so-called all-stage resistance is detected at the seedling stage and is therefore also referred to as seedling resistance. Nevertheless, seedling resistance is in general expressed throughout all growth stages, leading to resistance in the seedling stage as well as in adult plants. It is monogenetically inherited, qualitatively expressed, and the underlying major genes are only effective against a subset of races (Chen, 2005; Feng et al., 2018). Thus, it mainly follows the gene-for-gene concept, in which the resistance depends on a specific genetic interaction between the host-resistance genes and the avirulence genes of the pathogen (Flor, 1971). Effectors produced by the pathogen are recognized by nucleotide binding site-leucine rich repeat (NB-LRR) proteins, predominately encoded by corresponding plant resistance genes (Flor, 1956; Juliana et al., 2018). This results in an effector-triggered immunity that usually initiates a hypersensitive response leading to a localized programmed cell death preventing further colonization, e.g., in the case of *Yr5, Yr7, Yr10*, and *YrSP* (Heath, 2000; Jones and Dangl, 2006). The use of race-specific resistance in plants is common in wheat, leading to a breakdown of major resistance genes according to the so-called boom-and-bust cycles (McDonald and Linde, 2002a). To date, most race-specific resistance genes against stripe rust, e.g., *Yr10, Yr24*, and *Yr27* have been overcome by virulent races leading to the demand for more durable resistance (Kolmer, 2005; Hovmøller et al., 2017; Wang and Chen, 2017). Adult plant resistance (APR), effective at later growth stages, is quantitatively inherited and based on minor genes encoding various resistance responses, which are not restricted to specific pathogen races (Krattinger and Keller, 2016). Thus, APR does not follow the gene-for-gene interaction and is generally considered as durable. A special type of APR to stripe rust is the high-temperature adult plant (HTAP) resistance that is additionally affected by temperature (Chen, 2013). However, the mechanisms of such durable resistances include an increased latency period, reduced uredinia size, reduced infection frequency, and reduced spore production to inhibit fungal infestation (Rosewarne et al., 2013). To improve the general stripe rust resistance in commercial cultivars, more genes and useful genetic markers are needed for increasing the level and durability of resistance by combining HTAP resistance with seedling resistance.

In the context of detecting new resistance genes and QTL, molecular markers are no longer the limiting factors due to the availability of high-throughput marker systems (Mammadov et al., 2012; Chen et al., 2014; He et al., 2014; Bayer et al., 2017; Cui et al., 2017), but rather the genetic variation present in the respective experimental populations that merge genomes of diverse founders *via* designed crosses (Asimit and Zeggini, 2010; Gibson, 2012). Such experimental populations are traditionally derived from crosses of two contrasting parents. Thus, only two alleles at a given locus segregate in such bi-parental populations (Han et al., 2020). In contrast, the strategy of multiparent advanced generation intercross (MAGIC) populations is to interrogate multiple alleles to achieve increased recombination and mapping resolution (Cavanagh et al., 2008). Prior to developing such MAGIC populations, founder lines have to be selected based on genetic and/or phenotypic diversity. The development itself includes three steps: (1) Selected parents are crossed with each other to form a broad genetic base. (2) To increase recombination events, advanced intercrosses among the mixed lines are performed. (3) Recombinant inbred lines (RILs) are created *via* single seed descent or by doubled haploid production (Huang et al., 2015). This procedure results in a high number of recombination events enhancing the mapping resolution (Han et al., 2020).

The Bavarian MAGIC wheat population (BMWpop) is one of the only two German MAGIC wheat populations, which are mainly based on adapted German elite cultivars (Sannemann et al., 2018; Stadlmeier et al., 2018). It captures 71.7% of the allelic diversity present in the German wheat breeding gene pool (Stadlmeier et al., 2018). Thus, the BMWpop provides a greater potential to detect new QTL for resistance to important fungal pathogens as has been shown for powdery mildew, septoria tritici blotch, tan spot, leaf rust, and additional important agronomic traits (Stadlmeier et al., 2018, 2019; Rollar et al., 2021). The objectives of the present study were to (i) phenotype the BMWpop for quantitative and qualitative stripe rust resistance in multi-environment field trials and an extensive seedling test and to (ii) map QTL for these resistances to develop closely linked molecular markers suitable for marker-assisted selection (MAS).

## Materials and Methods

### Plant Material

The study is based on the multiparental BMWpop comprising eight elite wheat cultivars (Stadlmeier et al., 2018). It consists of 394 diverse F_6 : 8_ RILs, which were derived from a simplified eight founder MAGIC mating design with additional eight-way intercrosses. The founders “Event”, “Bayp4535”, “Potenzial”, “Bussard”, “Firl3565”, “Format”, “Julius”, and “Ambition” originated from German and Danish wheat breeding programs and were selected on the criteria described by Stadlmeier et al. (2018). Detailed information about the development and the genetics of the BMWpop were described by Stadlmeier et al. (2018).

### Phenotypic Assessment of Stripe Rust Resistance in Field Trials

Six field trials were performed, each using a randomized incomplete block design with two replications at three locations in Germany: Quedlinburg (QLB, 51° 46′ 21.45 ″N 11° 8′ 34.8″ E) in Saxony-Anhalt, Soellingen (SOE, 52° 5′ 45.506 ″N 10° 55′ 41.711″ E) and Lenglern (LEN, 51° 35′ 47.53 ″N 9° 51′ 39.118″ E) in Lower Saxony. The 394 RILs, the eight founders, and the susceptible standard “Akteur” were evaluated for stripe rust resistance in double rows under natural disease epidemics in SOE (2017 and 2018) and LEN (2018 and 2019). In QLB, entries were sown in 2016/2017 and 2017/2018 in double rows of 1 m length with 30 plants per row and a spacing of 0.2 m between rows. Additional spreader plots with susceptible varieties were sown in regular intervals of every third plot. To ensure uniform infestation, the spreader plots were artificially inoculated in spring at the time of stem elongation (BBCH30, Meier, 2018) using the highly virulent *Puccinia striiformis* isolate Warrior + YR27 (Supplementary Table 1). For this, a spore suspension of 10 mg uredospores in 100 ml Isopar M (ExxonMobil Chemical Company, USA) was applied in a total amount of 10 ml suspension per m^2^, using a hand-held spinning disc sprayer (Bromyard, UK). Phenotyping of the trials was carried out by scoring the average percentage of infected leaf area of the second and third youngest leaf in two rows at two to four subsequent dates according to Moll et al. (2010). Scoring started at the time of clearly visible disease symptoms on spreader plots and/or when leaves of the susceptible standard “Akteur” showed ≥10% diseased leaf area and was conducted in 1-to-2-week intervals.

### Phenotypic Assessment of Stripe Rust Resistance in Seedlings

All RILs, the parental lines, and the susceptible standard “Akteur” were evaluated for resistance at the seedling stage in a detached leaf assay (Lück et al., 2020). Seedlings were grown in 77-cell propagation trays with mixed potting soil (Gebr. Patzer GmbH Co KG, Germany) using a randomized complete block design with four replications. Water agar (7 g L^−1^) containing 45 mg L^−1^ benzimidazole (Sigma-Aldrich Chemie GmbH, Germany) for delaying senescence of leaf segments, was dispensed in 4 x 10 mL aliquots into non-sterile 4-well polystyrene plates (8 × 12 × 1 cM, Greiner Bio-One GmbH, Germany). Ten days after sowing, when the second leaf was fully developed, 2.5 cM sections were cut from the middle of the primary leaves and placed into the plates according to the initial randomization. White polytetrafluoroethylene frames (eMachineShop, NJ, USA) were used to fix the leaves. Inoculation was performed by an infection tower with the swirling duration of 3 s and settling time of 3 min (Melching, 1967). Due to space restrictions, the plates were divided into two infection groups per replication. Each group was inoculated with stripe rust isolate Warrior + YR27 using a mixture of 50 mg uredospores and white clay (1:1 w/w, VWR International GmbH, Bruchsal, Germany) after the application of a 0.01% Tween 20 (Sigma-Aldrich) solution to support adhesion. For the first 24 h of incubation, the plates were covered by wet cotton paper, and placed into a climate cabinet at 7°C to support spore germination. Next, inoculated leaf segments were incubated in a growth chamber at night/day temperatures of 16°C/18°C with additional lighting (16 h/8 h day/night) for 15 days. Quantitative scoring was conducted using the high-throughput phenotyping platform “Macrobot” (Lück et al., 2020). Digital images with a resolution of 20 megapixel and four wavelengths between 315 nm and 750 nm (UV, blue, green, and red) were taken automatically from every plate. Subsequently, the leaf area was calculated and compared to the area of uredospore pustules for analyzing the percentage of infected leaf area (Pi) using the software HawkSpex® (Fraunhofer IFF, Germany). Additionally, all entries were visually evaluated for infection type (IT) using a 0–4 scale (McIntosh et al., 1995). To generate metric data, original IT data were converted to a 0–10 linear disease scale, modified according to Zhang et al. (2014), as below: 0, 0, N, −1, 1, +1, −2, 2, +2, −3, 3, +3 were coded as 0, 0.5, 0.75, 1, 2, 3, 4, 5, 6, 7, 8, and 9, respectively. The values IT −4 and 4 were coded as 10.

### Data Analysis

The multiple scorings of the percentage of Pi in field trials were taken to calculate the area under the disease progress curve (AUDPC) and the average ordinate (AO) (Moll et al., 1996) for each RIL according to Rollar et al. (2021). For subsequent statistical analysis, only the AO values were used. Different year-location combinations of all trials were referred to as “environment”. The analyses of all phenotypic data were carried out using *proc mixed* of the software package SAS 9.4 (SAS Institute Inc., NC, USA). To apply a mixed linear model, a log_10_ data transformation of the AO, IT, and Pi values was performed. The factors, such as genotype, environment, and the genotype × environment interaction of field data, were set as fixed effects, while the design effects of replication and block were set as random. To obtain variance components for calculation of the broad-sense heritability, all model parameters were set as random. Heritability was estimated on a progeny mean basis using the formula according to Hallauer et al. (2010):


$$\begin{array}{l} h^{2}=\frac{V_{G}}{\frac{V_{E}}{re}+\frac{V_{GE}}{e}+V_{G}} \end{array}$$

Where *V*_*G*_ is the genotypic variance, *V*_*E*_ is the environmental variance, *V*_*GE*_ is the genotype × environment variance, and *r* and *e* are the number of replicates and environments, respectively. For analyzing IT and Pi scores from the seedling test, the following formula was used:


$$\begin{array}{l} y_{ijk}=\mu +g_{i}+r_{j}+l_{k}\left(r_{j}\right)+e_{ijk} \end{array}$$

Where *y*_*ijk*_ is the trait observation, μ is the overall mean, *g*_*i*_ is the fixed effect of the genotype, *r*_*j*_ is the fixed effect of the replication, *l*_*k*_ is the random effect of the infection group nested in the replication, and *e*_*ijk*_ is the random residual error. Variance components were obtained by setting the genotype as random to calculate the repeatability as the ratio of the genotypic variance and the sum of the genotypic and the residual error variance divided by the number of replications. For each trait, least square means (ls means) were calculated and used for subsequent QTL analysis.

### QTL Mapping

The BMWpop and the parental lines were genotyped using the 15K + 5K Infinium® iSelect® array (TraitGenetics, Germany) containing 17,267 single nucleotide polymorphisms (SNPs). The preparation of genotypic data and the construction of the linkage map used for QTL mapping were described in detail by Stadlmeier et al. (2018). QTL mapping was performed using the R (x32 3.2.5) package mpMap V2.0.2 (Huang and George, 2011; R Core Team, 2017). To conduct simple interval mapping (SIM), founder probabilities were calculated using the function “mpprob”. To determine the parental origin of an allele, the threshold was set to 0.7. For SIM, a genome-wide significant threshold of α < 0.05 was calculated for each trait. The thresholds were obtained from permutation of phenotypic data with 1,000 simulation runs (Churchill and Doerge, 1994). QTL detection was performed using the function “mpIM”, implemented in the mpMap package (Huang and George, 2011). Phenotypic variance explained by individual QTL and additive QTL effects were estimated separately using the categorical allele information of the founders. A QTL support interval (SI) was defined as the map interval surrounding a QTL peak at a -log_10_(*p*) drop of one unit.

To compare QTL identified in the present study with previously described QTL, overlapping QTL were merged based on the support interval. Databases of the Triticeae Toolbox (https://triticeaetoolbox.org/wheat/genotyping/marker_selection.php), GrainGenes (https://wheat.pw.usda.gov/GG3/), as well as CerealsDB (https://www.cerealsdb.uk.net/cerealgenomics/CerealsDB/axiom_download.php) were used to obtain marker information. Physical positions were obtained by nucleotide BLAST (BLAST-n) of the marker sequences against the reference sequence RefSeq v1.0 (Appels et al., 2018) using the database of 10+ Genome Project (https://webblast.ipk-gatersleben.de/wheat_ten_genomes/, Deng et al., 2007). BLAST hits were considered as significant if the percent identity was greater than 95% and only the best hit was taken if multiple BLAST hits were detected (Gao et al., 2016). The start and end positions of peak marker sequences preceded by the chromosome name were taken to the URGI database to obtain functional gene annotations available from IWGSC (https://wheat-urgi.versailles.inra.fr/Seq-Repository/Annotations). Furthermore, a fixed chromosomal region of ± 500 kb on both sides of the QTL peak markers was examined for additional gene annotations and the output retrieved from URGI database was listed. Sequences of the closest related species, *Triticum urartu* (A-genome donor) and *Aegilops tauschii* (D-genome donor), were considered for the detection of orthologous genes.

## Results

### Phenotypic Assessment

**Figure 1:**
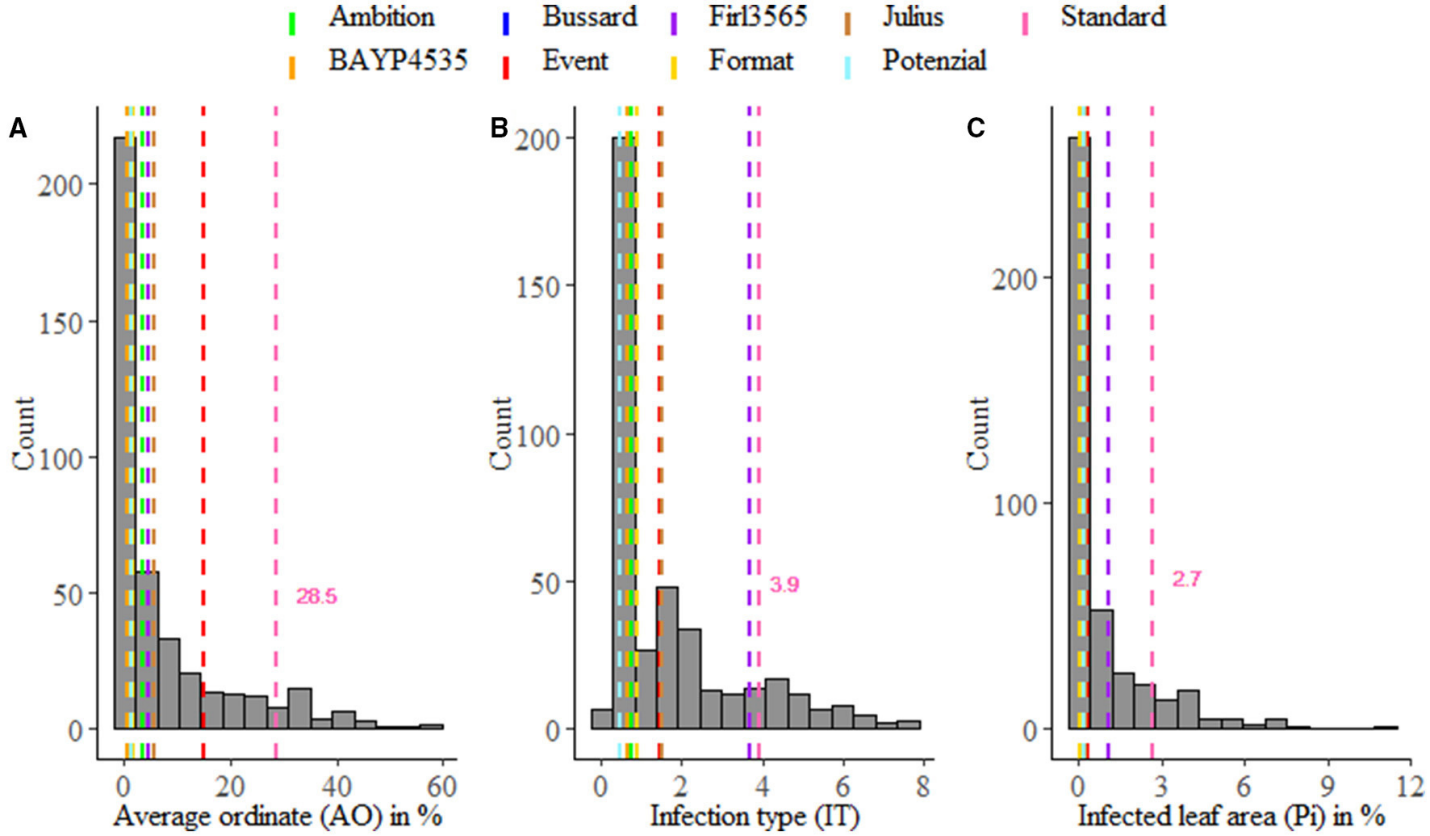
Averaged phenotypic distribution of resistance to *Puccinia striiformis* for field trials **(A)** and seedling test **(B,C)**. Performance of the parental lines and the susceptible standard cv. “Akteur” is shown as vertical dashed lines.

Stripe rust infestation of field trials was highly correlated between the year-location combinations (Supplementary Figure 1). Pearson's correlation calculations between the different environments showed only slight differences with high correlations between *r* = 0.75 and *r* = 0.86 (*p* < 0.001). A high heritability of *h*^2^ = 0.94 was calculated (Table 1). The mean phenotypic distribution of AOs was right skewed with 266 RILs showing an AO smaller than 5% (Figure 1A). However, the mean distribution ranging between 0.4 and 58.1% (mean 8.0%) diseased leaf area and single maximum AO scores up to 98.1% were observed within the population (Figure 1A, Table 1). Six of eight founders showed mean AOs below 5%, resulting in a nonsignificant difference (*p* < 0.05) from the progeny mean. Founders “Bayp4535” and “Event” were identified as the most resistant (0.7%) and most susceptible (15.1%) parental lines to stripe rust, respectively. The analysis of variance showed significant differences concerning the genotype, environment, and the interaction between genotype and environment (Table 2).

**Table 1:** Descriptive statistics of raw data and heritability/repeatability for field trials (AO) and seedling test (IT and Pi).

**Trait^a^**	**Mean founders**	**Mean population**	**Min^b^**	**Max^c^**	**SE^d^**	**CV^e^**	***h*^2^/*rep***
AO [%]	4.23	8.04	0	98.13	0.21	182.98	0.94^f^
IT [1-10]	1.28	1.72	0	10.00	0.06	129.08	0.76^g^
Pi [%]	0.22	0.92	0	25.00	0.07	185.74	0.58^g^

a *Average ordinate (AO), infection type (IT), infected leaf area (Pi)*.

b *Minimum*.

c *Maximum*.

d *Standard error*.

e *Coefficient of variance*.

f *Broad-sense heritability (h^2^)*.

g *Repeatability (rep)*.

**Table 2:** Analysis of variance of log_10_-transformed data for leaf rust severity evaluated in field trials (AO) and seedling test (IT and Pi).

**Trait^a^/factor**	**DF^b^**	***F* value**	***P* value**
**AO**			
Genotype	402	58.16	<0.0001
Environment	5	101.57	<0.0001
Genotype × environment	2009	1.99	<0.0001
**IT**			
Genotype	402	4.29	<0.0001
Replication	3	1.54	0.3369
**Pi**			
Genotype	402	2.52	<0.0001
Replication	3	1.80	0.2917

a *Average ordinate (AO), infection type (IT), infected leaf area (Pi)*.

b *Degrees of freedom*.

For IT and Pi assessed in the seedling inoculation test, the phenotypic data revealed a high degree of resistance (Figures 1B,C). Phenotypic distributions of IT and Pi were strongly right skewed, with 287 and even 388 RILs showing IT values smaller than 2 and Pi values below 5%, respectively. The average IT ranged from 0.1 to 7.8 (mean 1.7). For Pi, the disease severity was on average between 0 and 11.1% (mean 0.9%). Maximal scores of 10 (IT) and 25% (Pi) were observed (Table 1). The population mean for IT was not significantly different from the mean of the parental lines, while a significant difference between the population and founder mean for Pi was observed. For IT and Pi, respectively, the parental lines “Potenzial” and “Bayp4535” turned out to be the most resistant. “Firl3565” was the most susceptible founder in the seedling inoculation test. Pearson's correlation displayed a high correlation coefficient between both traits (*r* = 0.82; Supplementary Figure 2C). The traits IT and Pi and the scoring of AO showed moderate correlations of *r* = 0.63 and *r* = 0.46 (Supplementary Figures 2A,B). For both traits, a significant effect of the genotype was observed. Repeatability of IT was high with *rep*(IT) = 0.76, while a moderate repeatability for Pi was calculated (*rep*(Pi) = 0.58, Table 1).

### QTL Mapping

Overall, SIM revealed 21 QTL located on chromosomes 1A, 1D, 2A, 2B, 2D, 3B, 3D, 6A, and 7D. Eight of these were detected based on field data averaged over six environments, seven QTL were found for IT, and six QTL for Pi (Table 3, Supplementary Table 2).

**Table 3:** QTL for resistance to *Puccinia striiformis* in the BMWpop detected in field trials (AO) and seedling tests (IT and Pi).

**Trait**	**Chr.^a^**	**Pos.** **[cM]^b^**	**SI [cM]^c^**	***P* value**	***R* ^2^ ^d^**	**Eff (A)^e^**	**Eff (B)^e^**	**Eff (C)^e^**	**Eff (D)^e^**	**Eff (E)^e^**	**Eff (F)^e^**	**Eff (G)^e^**	**Eff (H)^e^**
**AO**													
No. Env.^f^													
5	1A	16.37	0-34	2.47E-09	0.23	na	+0.97	−1.98	na	+2.02	−0.94	−0.06	na
2	1D	62.37	51-76	1.18E-05	0.06	−0.71	+0.70	+1.24	−1.76	+1.63	+0.31	−1.78	+0.38
4	2B	105.57	101-182	5.17E-13	0.20	+1.84	−1.38	na	na	na	−1.45	+0.35	+0.60
5	2B	163.5	158-167	1.33E-18	0.29	na	−1.27	na	na	na	+0.14	+1.13	na
3	3B	218.05	212-225	2.09E-05	0.07	+0.12	−0.97	−1.20	+2.21	+1.71	+1.38	−1.11	−2.17
4	3D	13.94	5-62	1.53E-05	0.01	+1.13	−0.49	na	−1.13	−0.53	na	na	na
6	6A	259.48	258-264	1.75E-23	0.16	−0.10	+1.80	+1.10	−1.28	+1.10	na	na	−2.62
3	7D	19.64	12-30	2.16E-06	0.08	na	+2.31	na	na	−0.57	−1.95	+0.07	0.12
**IT**													
	1A	11.77	0-34	6.14E-09	0.11	na	+0.53	−0.88	na	+1.56	−0.70	−0.49	na
	1A	210.75	197-215	0.0235	0.06	+0.45	−0.75	+1.55	+1.83	−0.73	−0.57	−1.00	−0.81
	2A	0.5	0-13	0.0039	<0.01	+0.19	−0.83	+1.22	−0.98	na	na	+1.22	−0.83
	2A	32.16	21-44	0.0377	0.01	+1.10	+0.05	−0.19	−0.10	−0.38	−0.41	+0.02	−0.05
	2B	163.5	155-167	1.33E-18	0.16	na	−0.82	na	na	na	0.25	0.56	na
	2D	161.57	144-166	0.0426	0.09	−0.03	na	na	na	+1.14	na	na	−1.10
	6A	259.98	258-263	6.57E-23	0.16	−0.15	+1.14	+0.98	−1.19	+0.88	na	na	−1.66
**Pi**													
	1A	204.48	191-215	0.0470	0.08	+0.22	−0.65	+1.37	+1.57	−0.69	−0.61	−0.63	−0.63
	2A	1.51	0-13	0.0041	<0.01	+0.73	−0.16	−0.22	−0.06	na	na	−0.10	−0.18
	2B	163.5	155-169	1.33E-18	0.12	na	−0.78	na	na	na	+0.29	0.50	na
	2B	197.5	184-217	8.11E-08	0.05	na	−0.54	na	na	+0.53	na	na	na
	2D	161.57	144-166	0.0426	0.07	−0.07	na	na	na	+1.10	na	na	−1.03
	6A	259.98	258-265	6.57E-23	0.10	−0.62	+0.76	+0.50	+0.38	+0.40	na	na	−1.40

a *Chromosomal position of QTL*.

b *Position of peak marker based on the study by Stadlmeier et al. (2018)*.

c *Support interval*.

d *Proportion of phenotypic variance explained by a single QTL*.

e *Additive effects (±) of the founders Event (A), Bayp4535 (B), Ambition (C), Firl3565 (D), Format (E), Potenzial (F), Bussard (G), and Julius (H) relative to the population mean. Shown values are back-transformed to the original trait scale*.

f *Number of single environments in which a QTL was detected. Founder effects were reported as not available (na) if none of the RILs reached the probability threshold*.

The phenotypic variance (*R*^2^) explained by the individual QTL detected in field trials ranged between 1 and 29%, with SI from 6 cM to 81 cM. The three strongest QTL, explaining 23, 20, and 29% of *R*^2^, were located on chromosomes 1A and 2B with peak markers at 16 cM, 106 cM and 172 cM, respectively. “Ambition”, “Potenzial”, and “Bayp4535” contributed to the largest allelic effects of these QTL, reducing disease severity (AO) by 2, 1.5, and 1.3%. Another QTL detected on chromosome 6A (at 259 cM) explained 16% of the phenotypic variance with “Julius” as the most resistant founder line, reducing the Pi by 2.6%. On chromosomes 1A, 3B, and 7D, additional three QTL were detected at positions 62, 218, and 20 cM, respectively. The QTL accounted for 6% to 8% of stripe rust variation, while cv. “Bussard”, “Julius”, and “Potenzial” contributed to the largest allelic effects reducing the Pi by 1.8, 2.2, and 2.0%, respectively. The remaining QTL on chromosome 3D (4 cM) explained 1% of the phenotypic variance with “Firl3565” contributing to the highest allelic effect (-1.1%). All QTL detected over the mean of six environments were also identified by analyzing each environment separately (Supplementary Table 2). Hence, QTL located on chromosomes 1A, 1D, 2B, 3B, 3D, 6A, and 7D were identified in five (1A), two (1D), four (2B), five (2B), three (3B), four (3D), six (6A), and three (7D) environments, respectively (Table 3). However, on chromosome 4A, a QTL with a support interval (SI) between 159 cM and 200 cM was detected in LEN19, QLB18, QLB19, and SOE19, which was no longer significant when mean AO values across all environments were used (Supplementary Table 2).

For IT, the phenotypic variance explained by the seven QTL ranged from 1 to 16% with SIs between 5 and 34 cM (Table 3). QTL on chromosomes 2B and 6A accounted for the highest *R*^2^, i.e., 16% each with peak markers at 164 cM and 260 cM, respectively. The founders “Bayp4535” and “Julius” reduced disease severity by 0.8 and 1.7 IT scores, respectively, contributing to the largest allelic effects. On chromosome 2D, one QTL was detected at 162 cM, explaining 9% of the phenotypic variance. A maximum effect of −1.1 IT scores was detected for the allele derived from cv. “Julius”. Furthermore, two QTL were detected on chromosome 1A explaining 11% (at 12 cM) and 6% (at 211 cM) of the phenotypic variance. The cv. “Ambition” and “Julius” contributed to the highest allelic effect (−0.9 and −0.8 IT scores). Two QTL located on chromosomes 4D explained only 1% of the phenotypic variance each and were mapped at 1 cM and 32 cM.

QTL analysis of Pi values revealed six individual QTL with *R*^2^ ranging from less than 1 to 12%. The SIs varied between 7 and 33 cM. QTL regions on chromosomes 1A, 2A, 2B, 2D, and 6A overlapped with QTL regions detected for IT (Table 3). The *R*^2^ values of 12% (2B), 7% (2D), 10% (6A), 8% (1A), and <0.1% (2A) were calculated for individual QTL. The maximum reducing effects of each QTL for Pi ranged from 0.2 to 1.4%, contributed from different founders. Additionally, one QTL was detected on chromosome 2B at 198 cM, accounting for 5% of the phenotypic variance. A maximum effect of −0.5% was detected for the allele derived from the cv. “Bayp4535”.

Based on SIs of 21 QTL detected in total for AO, IT, and Pi, 13 main QTL regions were derived, i.e., those detected for all estimated traits (Supplementary Figure 3, Table 4). *In silico* annotations of peak markers revealed seven genes with known functions partly involved in resistance. Marker *wsnp_Ex_c6488_11266589* on chromosome 1A referred to CRS1-YhbY of *A. thaliana*, belonging to the chloroplast RNA splicing and ribosome maturation (CRM) domain-containing proteins. A dehydrogenase E1 component and a serine carboxypeptidase-like 19 were identified for peak markers for *QYr.jki-2A.1* and *QYr.jki-2A.2* on chromosome 2A. Markers *RAC875_c1226_652* and *AX-94388449* on chromosome 2B referred to BST_chr2B_nlr_143 and a formin-like protein 3, respectively. For the peak markers for *QYr.jki-2D* on chromosome 2D and *QYr.jki-3B* on chromosome 3B, GATA transcription factor 28 and a dual specificity phosphatase-catalytic domain were annotated. In addition, a fixed chromosomal region of ± 500 kb around each peak marker was examined. *In silico* annotations revealed additional gene annotations of different function on both sides of each QTL peak marker (Supplementary Table 4). On average, 24 gene annotations were identified within an interval of ± 500 kb on each side of the peak markers, including leucine-rich repeats for peak markers *AX-95080900* and *RAC875_c38756_141* of the QTL *QYr.jki-1A.1, wsnp_Ex_c28149_37293173* of QTL *QYr.jki-1A.2*, and *BobWhite_c13373_250* of *QYr.jki-2A.1*. In addition, NB-ARC domains were detected in the interval of peak markers *AX-95080900* and *wsnp_Ku_c23598_33524490* of QTL *QYr.jki-1A.1, wsnp_Ex_c6488_11266589* of QTL *QYr.jki-1A.2, BobWhite_c13373_250* and *wsnp_Ku_c23598_33524490* of *QYr.jki-2A.1, AX-95177447* of *QYr.jki-2A.2, RAC875_c1226_652* of QTL *QYr.jki-2B.2, AX-94734962* of *QYr.jki-2D*, and *TA005377-1076* of *QYr.jki-7D*. Furthermore, protein kinase domains and/or ABC transporters were identified in the vicinity of peak markers *AX-95080900* and *RAC875_c38756_141* of QTL *QYr.jki-1A.1, BobWhite_c13373_250* and *wsnp_Ku_c23598_33524490* of *QYr.jki-2A.1*, and *AX-94526138* for QTL *QYr.jki-6A*. However, a minimum of four different resistance related gene annotations were identified in the interval of peak marker *AX 94388449* of the QTL *QYr.jki-2B.3*, while the maximum of 43 respective annotations were detected for *BobWhite_c13373_250* being the peak marker of QTL *QYr.jki-2A.1* (Supplementary Table 4).

**Table 4:** Quantitative trait loci (QTL) resistance to *Puccinia striiformis* merged over all evaluated traits.

**QTL**	**Chr.^a^**	**Peak markers for different traits**	**Determined by**	**Pos. [cM]^b^**	**SI [cM]^c^**	**Pos. RefSeq [bp]^d^**		**Adjacent *T. aestivum* gene**	**Orthologous gene**	**Identity**	**Functional annotation**
						**Start**	**End**				
*QYr.jki-1A.1*	1A	AX-95080900	Field trials/ Seedling test	11.77	0-34	11893447	11893547				
		RAC875_c38756_141		16.37		7335009	7335109	TraesCS1A01G017400LC			
*QYr.jki-1A.2*	1A	wsnp_Ex_c28149_37293173	Seedling test	204.48	191-215	547965888	547966088	TraesCS1A01G370800	TRIUR3_02949^e^	99.85	
									F775_06956^f^	95.27	
		wsnp_Ex_c6488_11266589		210.75		550613052	550613249	TraesCS1A01G376400	F775_01986^f^	98.66	CRS1-YhbY (CRM-domain)
*QYr.jki-1D*	1D	AX-94614313	Field trials	62.37	51-76	262248014	262248114	TraesCS1D01G294200LC			
*QYr.jki-2A.1*	2A	BobWhite_c13373_250	Seedling test	0.50	0-13	3962381	3962481	TraesCS2A01G010100	TRIUR3_01629^e^	97.70	Dehydrogenase E1 component
									F775_30864^f^	97.24	
		wsnp_Ku_c23598_33524490		1.51		3447394	3447594	TraesCS2A01G007800	F775_31644^f^	98.22	
*QYr.jki-2A.2*	2A	AX-95177447	Seedling test	32.16	21-44	18165504	18165604				Serine carboxypeptidase-like 19^*^
*QYr.jki-2B.1*	2B	RAC875_rep_c109207_706	Field trials	105.57	101-182	69015103	69015203	TraesCS2B01G108000			
*QYr.jki-2B.2*	2B	RAC875_c1226_652	Field trials/ Seedling test	163.5	155-169	157693534	157693634	TraesCS2B01G182800			BST_chr2B_nlr_143
*QYr.jki-2B.3*	2B	AX-94388449	Seedling test	197.5	184-217	576083328	576083428	TraesCS2B01G406800	TRIUR3_14851^e^	98.97	Formin-like protein 3^*^
*QYr.jki-2D*	2D	AX-94734962	Seedling test	161.57	144-166	636599900	636600000	TraesCS2D01G568600	F775_15392^f^	99.55	GATA transcription factor 28^*^
*QYr.jki-3B*	3B	BobWhite_c14365_59	Field trials	218.05	212-225	640059368	640059468	TraesCS3B01G404700	TRIUR3_12644^e^	98.84	Dual specificity phosphatase - catalytic domain
*QYr.jki-3D*	3D	Kukri_c3773_1450	Field trials	13.94	5-62	na	na				
*QYr.jki-6A*	6A	AX-94526138	Field trials, Seedling test	259.48	258-265	608502823	608502923	TraesCS6A01G598000LC			
		BS00067558_51		259.98		606439738	606439838	TraesCS6A01G391800	TRIUR3_27114^e^	98.15	
									F775_21380^f^	95.94	
*QYr.jki-7D*	7D	TA005377-1076	Field trials	19.64	12-30	13295533	13295582	TraesCS7D01G027100	TRIUR3_33401^e^	96.45	
									F775_32200^f^	100.00	

a *Chromosomal position of QTL*.

b *Position of peak marker based on the study by Stadlmeier et al. (2018)*.

c *Support interval*.

d *Position of peak marker in the reference sequence RefSeq v1.0*.

e *Triticum urartu*.

f *Aegilops tauschii*.

* *Information provided by https://www.cerealsdb.uk.net/cerealgenomics/CerealsDB/axiom_download.php*.

## Discussion

Stripe rust occurs worldwide and is one of the most important pathogens in wheat cultivation. Known stripe rust resistances are present in many cultivars; however, their effectiveness is limited to certain races within the rust population in accordance with the gene-for-gene hypothesis (Flor, 1971). The emergence and selection of virulent pathotypes and their broad distribution results in considerable intraspecific variations in rust populations (Zetzsche et al., 2019). This in general causes the breakdown of qualitative resistances just a few years after their release (McDonald and Linde, 2002b; Kolmer, 2005). Thus, a continuous effort in wheat breeding programs is required to obtain a high degree of resistance to stripe rust by combining qualitative resistance genes with major effects and more durable APR. In this respect, the use of MAGIC populations in various QTL mapping studies turned out to be a powerful tool to detect both qualitative and quantitative resistance genes to different pathogens and other economically important traits (Pascual et al., 2015; Sallam and Martsch, 2015; Sannemann et al., 2015; Stadlmeier et al., 2019; Rollar et al., 2021).

In this study, more than 68% of the 394 RILs showed resistance to *Pucchinia striiformis*. A possible explanation for this can be found in the nature of the founder lines, of which almost all showed a high level of resistance to *P. striiformis* (Figure 1) suitable for the registration of varieties. Phenotypic data with many 0-values can lead to non-normally distributed residuals and thus affect the estimation of QTL effects in a regression-based QTL analysis. However, in this study, the phenotypic data were log_10_-transformed to ensure a normal distribution of the residuals for interval mapping. Thus, the right skewed distribution of the original phenotypic data did not affect the QTL detection results. With an average correlation coefficient of *r* = 0.82, minor differences between the disease severities in the six analyzed environments were observed. Additionally, a high broad-sense heritability of *h*^2^ = 0.94 was calculated, which is in the range of previously published studies (Feng et al., 2018; Liu et al., 2018; Ma et al., 2019; Yang et al., 2019a). These results indicate that stripe rust resistance is highly heritable and that QTL detected in the different environments were less affected by the occurrence of different *P. striiformis* races and/or different environmental conditions (Feng et al., 2018). Correlation between field data and seedling test results were as follows: *r* = 0.63 for IT and *r* = 0.46 for Pi, which are higher than the already reported correlations for leaf rust (Gao et al., 2016; Rollar et al., 2021). However, this observation may indicate similar scorings for seedling and adult plant resistance.

A method for linkage mapping in a MAGIC population was applied first by Xu (1996) based on the regression methods of Haley and Knott (1992). This method was used and subsequently improved based on parent probabilities by Mott et al. (2000), resulting in HAPPY. On this base, Huang and George (2011) finally developed the “mpMap” package, which was used in this study, by following a mixed-model context and including environmental and pedigree effects in the analysis. There are two main advantages of MAGIC populations: (1) Due to the crossing design of MAGIC populations, an increased genetic variation and recombination rate are achieved and (2) due to the increased genetic variation, QTL detection can be performed with increased precision and resolution (Cavanagh et al., 2008; Bandillo et al., 2013; Holland, 2015; Stadlmeier et al., 2019; and Rollar et al., 2021). This also comes along with smaller linkage blocks, a higher accuracy, and smaller SIs (Li et al., 2005; Stadlmeier et al., 2019). Overall, simple interval mapping in this study detected 21 QTL, of which only one QTL showed SI ≤ 5 cM. Nevertheless, Stadlmeier et al. (2019) successfully demonstrated the detection of QTL with small SIs in the BMWpop, which was supported by similar findings in other advanced intermated populations (Balint-Kurti et al., 2007; Huang et al., 2010). In the present study, 19% of the detected QTL showed SIs < 10 cM, and an average SI of 23 cM was calculated. Compared to double haploid (DH) lines, MAGIC populations are not completely homozygous. This residual heterozygosity can lead to problems, as heterozygotes for some markers cannot be distinguished in genotyping (Huang et al., 2015). This is particularly the case for polyploids and genotyping-by-sequencing (GBS) approaches (Elshire et al., 2011; Cavanagh et al., 2013). However, the mean proportion of heterozygous allele calls per RIL was described as 0.8% in the BMWpop (Stadlmeier et al., 2018).

The 21 QTL detected for AO, IT, and Pi correspond to 13 distinct chromosomal regions (Table 4, Supplementary Figure 3). QTL identified using the ls means across the six environments were also identified in the analyses of single environments (Supplementary Table 2). Additionally, a QTL for AO on chromosome 4A was detected in LEN19, QLB18, QLB19, and SOE19, describing 6% of phenotypic variance on average. Although this QTL was no longer significant by analyzing mean AO values across all environments, it may be of importance since there seems to be a relation to a QTL for leaf rust (*QLr.jki-4A.2*) mapped in a previous study (Rollar et al., 2021). At 13 distinct chromosomal regions, each of the five QTL was detected at the adult plant and seedling stages only. In contrast, three QTL were common to both growth stages, indicating the presence of effective all-stage stripe rust resistance genes. In total, the 13 QTL regions were located on wheat chromosomes 1A, 1D, 2A, 2B, 2D, 3B, 3D, 6A, and 7D.

Peak markers of QTL were partially annotated to genes, known to be involved in resistance mechanisms of plants. It was described that several serine carboxypeptidase-like proteins (*QYr.jki-2A.2*) catalyze the production of plant secondary metabolites involved in herbivory defense and UV protection (Fraser et al., 2005). Mugford et al. (2009) also reported a possible contribution of serine carboxypeptidase-like proteins in the synthesis of acylate plant defense compounds (avenacins) in oats. Peak marker *wsnp_Ex_c6488_11266589* for *QYr.jki-1A.2* was annotated to CRS1-YhbY, with a CRM protein domain. It was shown that CRM domain-containing proteins isolated from maize contribute to RNA binding activity (Barkan et al., 2007). Such RNA binding proteins are involved in various important cellular processes and in posttranscriptional regulation of gene expression, respectively. Thus, the RNA binding proteins play an important role in plant immune response regulation against pathogens, as they allow for a quick response to biotic and abiotic stress stimuli (Woloshen et al., 2011). A similar finding is the GATA transcription factor 28 for marker *AX-94734962* on chromosome 2D. The GATA gene family is one of the most conserved families of transcription factors, playing a significant role in different aspects of cellular processes, e.g., in the abiotic stress signaling pathways (Gupta et al., 2017). The pyruvate dehydrogenase (E1) complex annotated for *BobWhite_c13373_250* on chromosome 2A is involved in two interacting levels of control in plant cells. The first level is subcellular compartmentation contributing to tricarboxylic acid cycle and fatty acid biosynthesis, while the second level is the control of gene expression (Tovar-Méndez et al., 2003). The mean linkage disequilibrium (LD) decay for the genome in the BMW population is 9.3 cM, thus, considering a fixed interval of ± 5 Mb on both sides of a peak marker resulted in an excessive number of gene annotations (Stadlmeier et al., 2018). In this study, the fixed interval was reduced to ± 500 kb (1 Mb) based on several other studies in which the region on either side of the peak marker of a QTL was reduced to 100 kb (flax) (You and Cloutier, 2020), 2 kb (wheat) (Juliana et al., 2018), 2 kb (wheat) (Muqaddasi et al., 2020), or 100 kb (rice) (Hussain et al., 2020). However, examination of this interval led to the annotation of several leucine-rich repeats, NB-ARC domains, kinase domains, and ABC transporters. While leucine-rich repeats and NB-ARC domains are mainly involved in race-specific resistance responses, quantitative race unspecific resistance genes appear to encode different proteins, such as ABC transporters, protein kinases, and hexose transporters (Ellis et al., 2014; Moore et al., 2015; and Periyannan et al., 2017).

For the majority of the QTL detected in this study, the effect magnitudes were rather small as a high fraction of the population was highly resistant indicating that major stripe rust QTL were common to the founder lines. Two QTL were detected on chromosome 1A based on both field and seedling test data (*QYr.jki-1A.1)* and on seedling test data (*QYr.jki-1A.2*) only. *QYr.jki-1A.1* is physically located in a region between 1.3 Mb and 12.5 Mb (Supplementary Table 3). To date, only one QTL for all-stage resistance to stripe rust was previously described in a similar region (Liu et al., 2018). *QYrMa.wgp-1AS* was mapped to the distal part of chromosome 1AS with the closest markers at 7.3 Mb (*IWB57448*) and 9.1 Mb (*IWB5441*). *IWB57448* was also detected as peak marker for *QYr.jki-1A.1* in this study (Table 4, Supplementary Table 3). Thus, the two QTL seem to be identical. *QYr.jki-1A.2* was physically located at the distal end of chromosome 1AL between 540 Mb and 593 Mb. In the same region, there are two QTL (*QYr.caas-1AL, QRYr1A.1*) for APR to stripe rust (Ren et al., 2012; Rosewarne et al., 2012). These QTL were mapped at around 551 Mb and 575 Mb, respectively, but both were inconsistently detected across several environments. Another QTL (*QYr.wsu-1A.2*) detected at the adult plant stage and associated with marker *IWA3215* was closely mapped to the distal end of *QYr.jki-1A.2* around 593 Mb (Bulli et al., 2016). However, Jighly et al. (2015) described a QTL for seedling resistance that corresponds to *QRYr1A.1* detected by Rosewarne et al. (2012) based on the linked DArT marker *wPt-6005*. Although *QYr.jki-1A.2* was only detected in the seedling test, relationships between the aforementioned QTL previously described and *QYr.jki-1A.2* based on physical positions might be possible.

On chromosome 1D, *QYr.jki-1D* was mapped in a large physical interval between 33 Mb and 366 Mb. However, the peak marker was located at 262 Mb. Furthermore, four QTL have been described at the distal end of chromosome 1DS, but none of these have been physically mapped near the region of *QYr.jki-1D* (Zwart et al., 2010; Vazquez et al., 2012; Hou et al., 2015; Naruoka et al., 2015). Maccaferri et al. (2015) reported the QTL *QYr.ucw-1 D* as a novel QTL independent of the aforementioned QTL. Its linked marker *IWA980* is physically mapped at 36.3 Mb and is thus within the SI of *QYr.jki 1D*, but still far away from our peak marker (Supplementary Table 3). Ren et al. (2012) identified a QTL (*QYr.caas-1D*) flanked by markers *Xgwm353* and *Xgdm33b* on chromosome 1DS in cv. “Naxos”, but no physical marker information is available for a closer comparison (Supplementary Table 3). The resistance gene *Yr25* was mapped on chromosome 1D and is one of the common *Yr* genes identified in European cultivars (McIntosh, 1988; Hovmøller, 2007). The stripe rust race Warrior + Yr27 used for inoculation in this study is virulent to *Yr25* (Supplementary Table 1). This may give hint that *QYr.jki-1D* does not refer to this resistance gene.

*QYr.jki-2A.1* and *QYr.jki-2A.2* were both detected on chromosome 2AS based on the seedling test. To date, three designated *Yr* genes (*Yr17, Yr56*, and *Yr69*) and several QTL have been described on the short arm of chromosome 2A (Bariana and McIntosh, 1993; Hao et al., 2011; Lowe et al., 2011; Agenbag et al., 2012; Vazquez et al., 2012; McIntosh et al., 2014; Hou et al., 2016; Liu et al., 2018). *QYr.jki-2A.1* was mapped between 3.1 Mb and 4.2 Mb, with peak markers at 3.4 Mb (Pi) and 3.9 Mb (IT, Table 4, Supplementary Table 3). Liu et al. (2018) located *QYrMa.wgp-2AS* around 2.7 Mb, corresponding to the region of *Yr17*, which was introgressed from *Aegilops ventricosa* to the hexaploid wheat line “VPM1” (Bariana and McIntosh, 1993). Based on the physical distance to our peak markers, it seems likely that *QYr.jki-2A.1* corresponds to *QYrMa.wgp-2AS* and/or *Yr17*, respectively (Table 4, Supplementary Table 3). The second QTL *QYr.jki-2A.2* was different from *QYr.jki-2A.1* as the peak marker was mapped at 18.2 Mb. Nevertheless, *QYr.jki-2A.2* was mapped in a large physical region from 5.7 Mb to 36.1 Mb, showing relationships with three QTL (*QYr.ufs-2A, QYr.uga-2AS, QYr.ucw-2AS*), as described previously. *QYr.ufs-2A* detected by Agenbag et al. (2012) was located in a region similar to *QYr.ucw-2AS* (Lowe et al., 2011) and *QYr.uga-2AS* (Hao et al., 2011). *QYr.ucw-2AS* was detected in an RIL population (“UC1110” × “PI610750”) and is flanked by markers *wPt-5839* and *Xwmc177*, of which the latter was mapped at 33.7 Mb (Lowe et al., 2011). *QYr.uga-2AS*, which was derived from cv. “Pioneer26R61”, was flanked by SSR markers *Xbarc124* (3.9 Mb) and *Xgwm359* (28.2 Mb) (Hao et al., 2011). Hence, all three QTL previously described are located in the chromosomal region of *QYr.jki-2A.2*, but further investigation is needed (Supplementary Table 3).

On chromosome 2B, QTL were detected based on field (*QYr.jki-2B.1*) and seedling test data (*QYr.jki-2B.3*) only, but also based on both data sets (*QYr.jki-2B.2*). QTL *QYr.jki-2B.1* was mapped to a large physical region between 69 Mb to 407 Mb, including the second QTL *QYr.jki-2B.2* (110.9 - 216.5 Mb). However, as the peak marker *RAC875_rep_c109207_706* was located at 69.0 Mb, *QYr.jki-2B.1* was designated separately and is assumed to be independent of *QYr.jki-2B.2* (Table 4, Supplementary Table 3). Chromosome 2BS is known to carry HTAP resistance that was detected in several wheat backgrounds (Ramburan et al., 2004; Guo et al., 2008; Carter et al., 2009; Chen et al., 2011). Chen et al. (2011) found *QYrid.ui-2B.1*, which was flanked by the markers *wPt-9668* and *Xgwm429*. The latter was physically mapped at 4.6 Mb proximal to the peak marker for *QYr.jki-2B.1*. As described by the authors, *QYrid.ui-2B.1* corresponds to two previously reported QTL: *QYr.sgi-2B.1* derived from cv. “Kariega” with the closest marker *Xgwm148* at 100.8 Mb (Ramburan et al., 2004) and *QYrlu.cau-2BS1* flanked by *Xwmc154* (36.4 Mb) and *Xgwm148* (100.8 Mb) (Guo et al., 2008). Based on these physical positions, *QYrid.ui-2B.1, QYr.sgi-2B.1*, and *QYrlu.cau-2BS1* appear to be located in the same region as *QYr.jki-2B.1* (Supplementary Table 3). For *QYr.jki-2B.2*, a similar conclusion can be drawn. In the study by Chen et al. (2011), a second QTL (*QYrid.ui-2B.2*) was identified, which was located in the same region as QTL *QYrlu.cau-2BS2Q* (Guo et al., 2008) and *Yrlo.wgp-2BS* (Carter et al., 2009). Together, the three QTL spanned a region from around 73.6 Mb to 448.7 Mb. The peak marker for *QYr.jki-2B.2* was mapped at 157.7 Mb, and thus is within the region of the three QTL described previously (Supplementary Table 3). The third QTL on chromosome 2BL (*QYr.jki-2B*.3) was detected for Pi values between 519 Mb and 724.5 Mb. Till date, there are seven designated *Yr* genes located on chromosome 2BL, of which *Yr5, Yr7*, and *YrSP* were already cloned between 615.8 Mb and 773.1 Mb (McIntosh et al., 2014; Marchal et al., 2018). Additionally, several QTL are described to be located at the long arm of chromosome 2B. One QTL was detected in the RIL population, “Camp Remy” × “Michigan Amber”, and flanked by SSR markers *Xgwm47* (685.8 Mb) and *Xgwm501* (672.1 Mb) (Boukhatem et al., 2002). Another QTL (*QYraq.cau-2BL*) derived from cv. “Aquileja” was mapped between the markers *Xwmc175* and *Xwmc332* corresponding to 670.6–739.4 Mb (Guo et al., 2008). Guo et al. (2008) described that *QYraq.cau-2BL* corresponds to QTL which were previously detected by Mallard et al. (2005) and Christiansen et al. (2006). These QTL in turn were assigned to the first-mentioned QTL detected by Boukhatem et al. (2002) and to resistance genes *Yr5* and *Yr7*, respectively (Supplementary Table 3). Although *QYr.jki-2B.3* seems to correspond to the aforementioned regions, the peak marker was mapped at 576.1 Mb, a physical distance of 94.5 Mb to the closest marker interval (Table 4, Supplementary Table 3). Thus, the relationship between *QYr.jki-2B.3* and the previously described QTL has still to be discussed. Furthermore, it is not clear whether *QYr.jki-2B.3* is related to the *Yr5, Yr7*, and *YrSP*.

*QYr.jki-2D* was mapped at the distal end of chromosome 2DL with the peak marker at 636.6 Mb. To date, there are six *Yr* genes (*Yr8, Yr16, Yr54, Yr55, Yr37*, and *YrCK*) known to be located on chromosome 2D. Unfortunately, no information on the physical positions is available for precise comparison. However, the APR gene *Yr16* was located in the centromeric region of chromosome 2D (Worland and Law, 1986; Ren et al., 2012), suggesting that this gene is different from *QYr.jki-2D*. Ren et al. (2012) reported a QTL on chromosome 2DL, flanked by the SSR marker *Xgwm539* (513.1 Mb) and *Xcfd44* (608.6 Mb). The authors assumed that this QTL is linked to two QTL as described previously, where both are closely linked to the marker *Xgwm349* (Suenaga et al., 2003; Melichar et al., 2008). This SSR marker is 7 bp apart from the peak marker of *QYr.jki-2D*. Hence, all three QTL may correspond to *QYr.jki-2D* (Supplementary Table 3).

On chromosome 3B, one QTL (*QYr.jki-3B*) was detected based on field trial data. The QTL SI spans a physical region from 581.3 Mb to 665.3 Mb, and is located on the long arm of chromosome 3B. There are many QTL previously reported that are partly summarized by Rosewarne et al. (2013) and Chen and Kang (2017). However, most of these are located on the short arm of chromosome 3B and do not correspond to *QYr.jki-3B*. In addition, the resistance genes *Yr4, Yr30*, and Yr57 were mapped on chromosome 3BS. Two QTL are detected on the long arm of chromosome 3B, *QYrex.wgp-3BL* (Lin and Chen, 2009) and *QYrid.ui-3B.2* (Chen et al., 2011). For both QTL, the SSR marker *Xgwm299* was reported as a flanking marker physically mapped at 804.8 Mb and does not correspond to the identified region of *QYr.jki-3B* (Supplementary Table 3). Recently, another QTL (*QYr-3BL*) was discovered in the durum wheat RIL population “Stewart” x “Bansi” flanked by the marker *IWB9451* (660.3 Mb) (Li et al., 2020). The authors associated this QTL with *Yr80*, a gene that is flanked by markers *KASP65624* and *KASP53113* spanning a physical region between 550.3 Mb and 605.4 Mb (Nsabiyera et al., 2018). Based on the physical positions, *QYr.jki 3B* may correspond to the resistance gene *Yr80*.

The quantitative trait locus *QYr.jki-3D* was mapped based on field data only. It is located at the distal end of chromosome 3DS between 19.8 Mb and 22.0 Mb. The two resistance genes *Yr49* linked to *Xgwm161* at 7.1 Mb, and *Yr66* linked to *IWB47165* at 2.6 Mb, as well as five QTL are described to be located on the arm of this chromosome (McIntosh et al., 2011, 2014; Basnet et al., 2013; Rosewarne et al., 2013). However, less marker information of QTL locations is available for precise comparison between *QYr.jki-3D* and QTL identified on chromosome 3DS by Boukhatem et al. (2002), Singh et al. (2000), and Basnet et al. (2013). Dedryver et al. (2009) found one QTL in cv. “Recital” flanked by the markers *Xbarc125* (174.8 Mb) and *Xgwm456* (282.5 Mb). Another QTL was mapped between 309.9 Mb and 357.1 Mb, far away from the region identified in this study (Yang et al., 2013). Thus, neither the QTL nor the *Yr* genes correspond to *QYr.jki-3D*, which therefore seems to be novel.

Based on the field and seedling test data conducted in this study, a QTL (*QYr.jki-6A*) was detected on chromosome 6AL, with peak markers at 606.4 and 608.5 Mb. There are three regions conferring resistance to stripe rust which are all closely linked to SSR marker *Xgwm617* (William et al., 2006; Lillemo et al., 2008; Vazquez et al., 2012), which is 2.1 and 4.2 Mb away from our peak markers. William et al. (2006) reported the presence of *QYr.cimmyt-6A*, which corresponds to the QTL found by Lillemo et al. (2008), both contributed by the cv. “Avocet”. It is likely that this QTL was derived from *Agropyron elongatum* due to a translocation in cv. “Avocet” (Lillemo et al., 2008). However, the third QTL (*QYrpl.orr-6A*) previously reported by Vazquez et al. (2012) was found in the RIL population “Stephens” × “Platte” and was also assigned to the QTL detected by Lillemo et al. (2008). A close relationship between these QTL and *QYr.jki-6A* can be assumed (Supplementary Table 3). Several additional QTL and major genes are reported to be located on chromosome 6A, including the resistance genes *Yr38, Yr42*, and *Yr81* (Marais et al., 2006, 2009; Prins et al., 2010; Cao et al., 2012; Rosewarne et al., 2012; Gessese et al., 2019). Unfortunately, the information provided was not sufficient to allow for further comparison.

The quantitative trait locus *QYr.jki-7D* based on data from field trials was located on the short arm of chromosome 7D. The QTL was physically mapped between 5.4 Mb and 29.4 Mb, with a position of the peak marker at 13.3 Mb. The five closest QTL already reported were linked to the SSR marker *Xgwm295* (53.6 Mb), which is 40.3 Mb apart from our peak marker (Ramburan et al., 2004; Navabi et al., 2005; Bariana et al., 2010; Yang et al., 2013). *Xgwm295* was found to be the closest microsatellite marker to the resistance complex *Lr34/Yr18* (Suenaga et al., 2003). In addition, Jighly et al. (2015) identified a QTL on chromosome 7DS linked to DaRT marker *wPt-668026*. The authors associated this QTL with the 7DS locus near the marker *Xbcd1438* described by Singh et al. (2000), which in turn was again associated with *Lr34/Yr18* (Jighly et al., 2015). This resistance gene has been functionally characterized and is already sequenced (Krattinger et al., 2009). However, due to the large distance between these QTL and the one detected in the present study, *QYr.jki-7D* seems to be a novel QTL (Supplementary Table 3).

The aim of this study was to use the Bavarian MAGIC wheat population to identify new sources of resistance to stripe rust, a fungal disease that causes devastating yield losses in wheat worldwide. The analyses resulted in 21 stripe rust resistance QTL that were confined to 13 distinct chromosomal regions. Eleven of these regions corresponded to QTL already described in previous studies. The increasing information on the physical map position of many stripe rust QTL, helped to infer the identity of the QTL found in the present study. Two putatively new QTL were identified on chromosomes 3D (*QYr.jki-3D*) and 7D (*QYr.jki-7D*). SNP markers linked to these regions may be converted into KASP markers suitable for MAS in wheat breeding programs (Wu et al., 2017; Yang et al., 2019b). This will enable stacking of the detected resistance loci to breed new varieties with an improved resistance to stripe rust. Additionally, data and information generated in the present study can be used for weighted selection (Bernardo, 2014).

## Data Availability Statement

The raw data supporting the conclusions of this article will be made available by the authors, without undue reservation.

## Author Contributions

LH and FO planned and managed the project. LH and MG provided and characterized all RILs. MG contributed to the analyses of the results. AS, FO, MG, LH, and VM contributed to the interpretation and discussion of the results. SR conducted the field screenings and seedling test, analyzed the data, and wrote the manuscript. All authors contributed to the article and approved the submitted version.

## Conflict of Interest

The authors declare that the research was conducted in the absence of any commercial or financial relationships that could be construed as a potential conflict of interest.

## Publisher's Note

All claims expressed in this article are solely those of the authors and do not necessarily represent those of their affiliated organizations, or those of the publisher, the editors and the reviewers. Any product that may be evaluated in this article, or claim that may be made by its manufacturer, is not guaranteed or endorsed by the publisher.

## Supplementary Material

Supplementary Figure 1 Pearson's correlation of stripe rust severity between different field trials. Diagonals are histograms for each environment (Lenglern LEN 2018-2019, Quedlinburg QLB 2017 2018, Söllingen SOE 2017 2018). ^***^ denotes significance at α = 0.001.

Supplementary Figure 2 Pearson's correlation (*r*) between averaged infection type (IT), infected leaf area (Pi) of seedling test and average ordinate (AO) of field trials **(A,B)**, as well as correlation between IT and Pi **(C)**. ^***^ denotes significance at α = 0.001.

Supplementary Figure 3 Simple interval mapping of resistance to *Puccinia striiformis* in field trials **(A)** and seedling test **(B,C)**. The x-axis shows the 21 wheat chromosomes. Positions are based on the genetic map, and the -log10(*p*) values of each marker are displayed on the y-axis (black line). The red horizontal line represents the significance thresholds. The seed index (SI) of the significant QTL detected in this study are colored in blue.

Supplementary Table 1 List of virulences and avirulences of Puccinia striiformis isolate, Warrior + Yr27 used in field trials and seedling test. Brackets indicate ambiguous results due to the differing symptom ratings between replications or moderate susceptibility (based on Zetzsche et al., 2019).

Supplementary Table 2 Complete information of the quantitative trait loci (QTL) for stripe rust resistance in BMW population, evaluated in field trials (AO) and seedling test (IT and Pi).

Supplementary Table 3 Comparison of the physical positions of the QTL identified in the present study (bold) with those reported previously.

Supplementary Table 4 List of gene annotations for peak markers ± 500,000 bp, shown as output retrieved from URGI database (https://wheat-urgi.versailles.inra.fr/Seq-Repository/Annotations).
