# Supplementary figures and images for: Quantitative Trait Loci Mapping of Adult Plant and Seedling Resistance to Stripe Rust (Puccinia striiformis Westend.) in a Multiparent Advanced Generation Intercross Wheat Population
Source: Front Plant Sci. 2021 Dec 23;12:684671. doi: 10.3389/fpls.2021.684671 (PMC8733622; doi:10.3389/fpls.2021.684671)

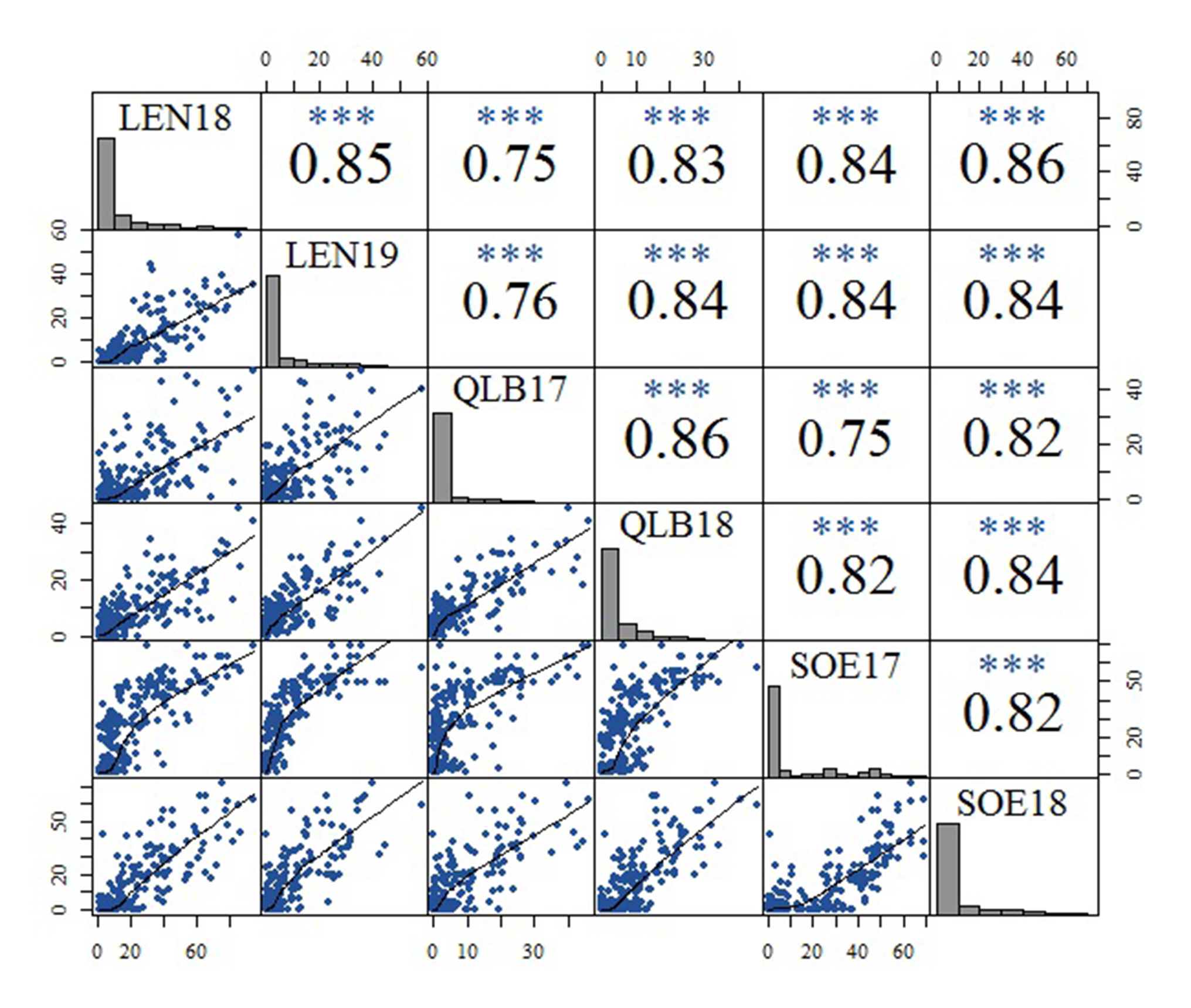

Supplement: Supplementary Figure 1 — Pearson's correlation of stripe rust severity between different field trials. Diagonals are histograms for each environment (Lenglern LEN 2018-2019, Quedlinburg QLB 2017 2018, Söllingen SOE 2017 2018). *** denotes significance at α = 0.001. [file Image_1.JPEG]

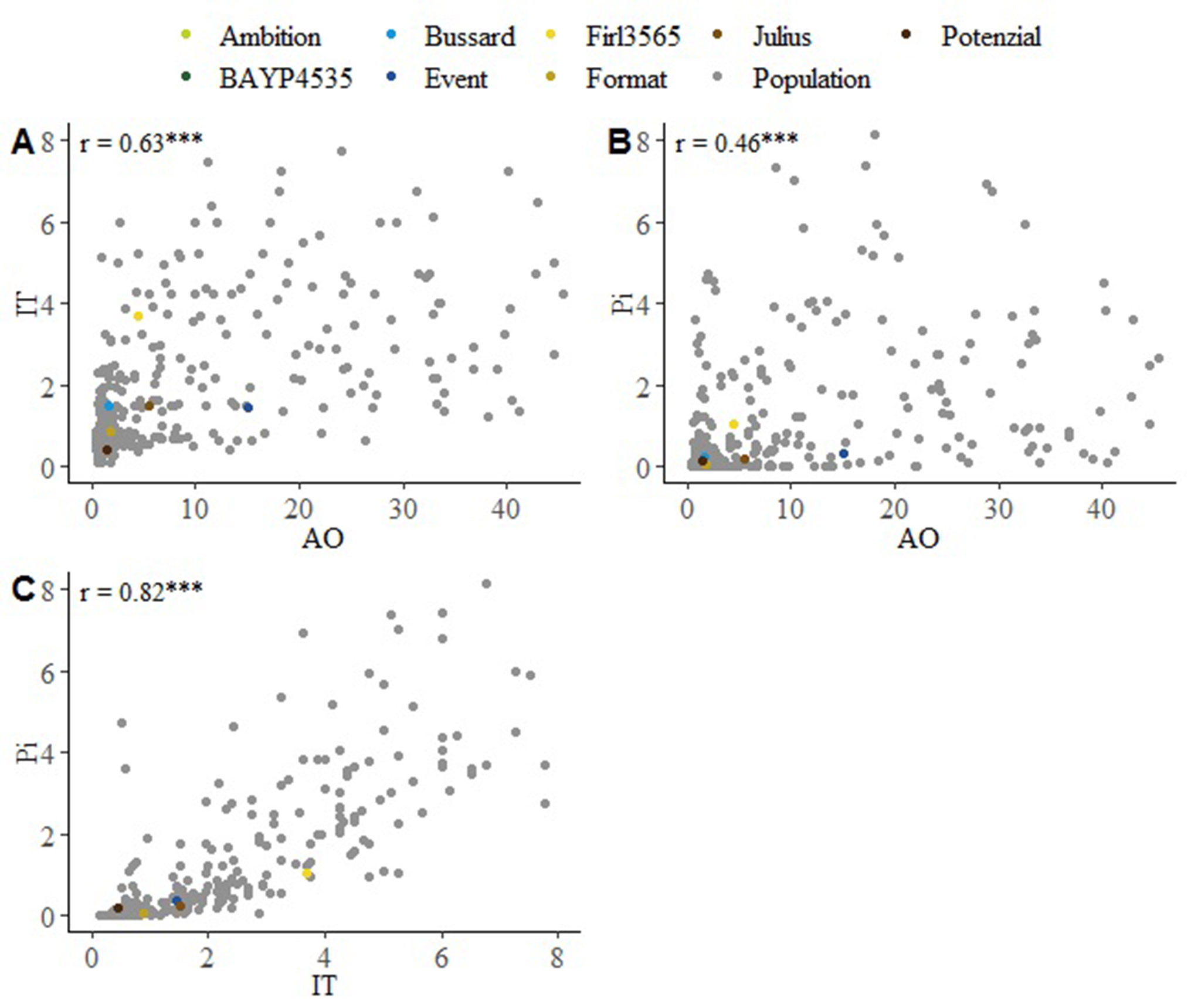

Supplement: Supplementary Figure 2 — Pearson's correlation (r) between averaged infection type (IT), infected leaf area (Pi) of seedling test and average ordinate (AO) of field trials (A,B), as well as correlation between IT and Pi (C). *** denotes significance at α = 0.001. [file Image_2.JPEG]

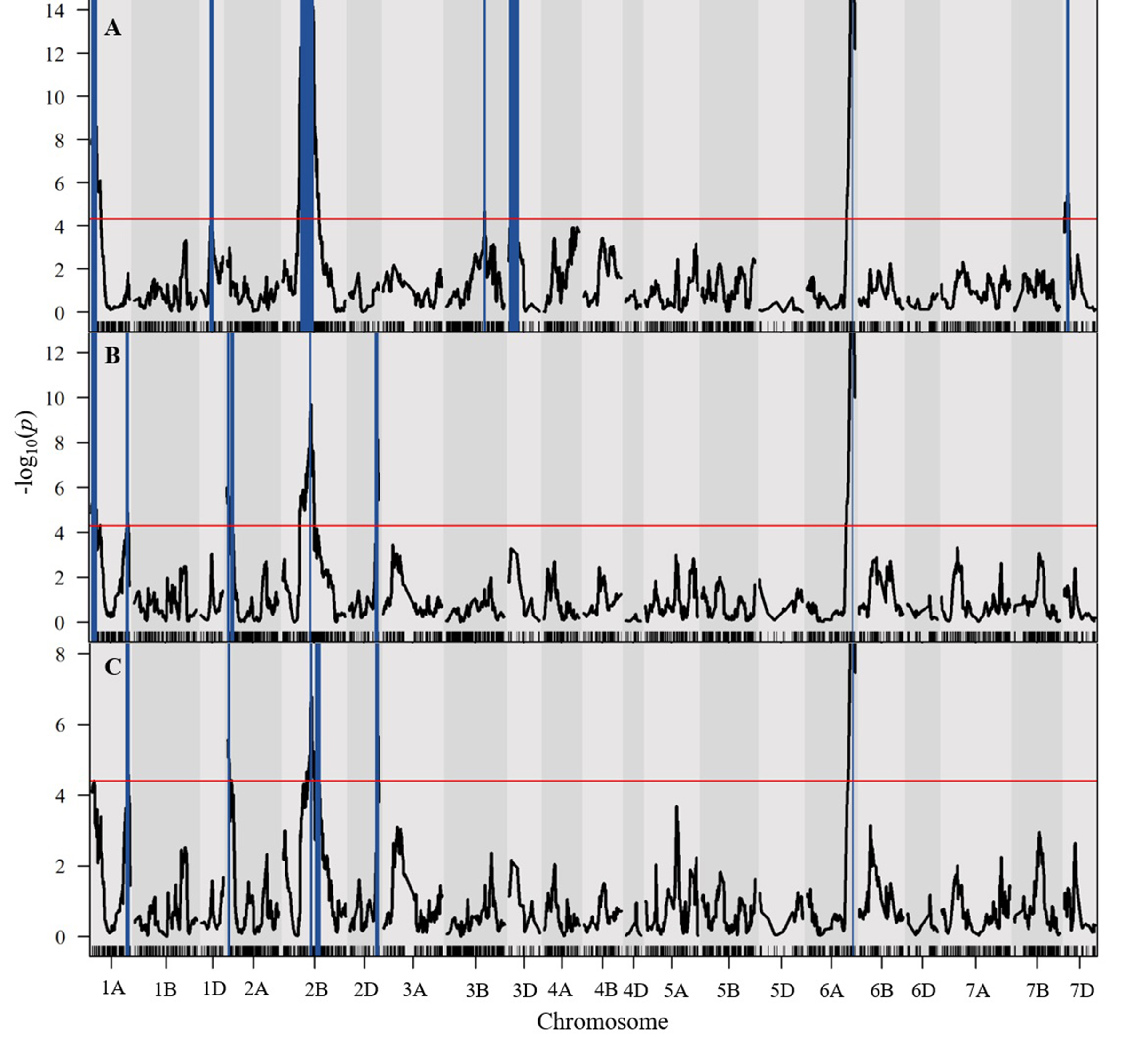

Supplement: Supplementary Figure 3 — Simple interval mapping of resistance to Puccinia striiformis in field trials (A) and seedling test (B,C). The x-axis shows the 21 wheat chromosomes. Positions are based on the genetic map, and the -log10(p) values of each marker are displayed on the y-axis (black line). The red horizontal line represents the significance thresholds. The seed index (SI) of the significant QTL detected in this study are colored in blue. [file Image_3.JPEG]
