# Supplementary material for: Quantitative Trait Loci Mapping of Adult Plant and Seedling Resistance to Stripe Rust (Puccinia striiformis Westend.) in a Multiparent Advanced Generation Intercross Wheat Population
Source: Front Plant Sci. 2021 Dec 23;12:684671. doi: 10.3389/fpls.2021.684671 (PMC8733622; doi:10.3389/fpls.2021.684671)
Supplement: Supplementary Table 1 — List of virulences and avirulences of Puccinia striiformis isolate, Warrior + Yr27 used in field trials and seedling test. Brackets indicate ambiguous results due to the differing symptom ratings between replications or moderate susceptibility (based on Zetzsche et al., 2019). [file Table_1.docx]

**Supplementary Table 1** List of virulences and avirulences of Puccinia striiformis isolate Warrior + Yr27 used in field trials and seedling test. Brackets indicate ambiguous results due to the differing symptom ratings between replications or moderate susceptibility (based on Zetzsche et al. 2019)

| *P. striiformis* f.sp. *tritici* | Virulence/avirulence | Virulences | Avirulences |
| --- | --- | --- | --- |
| Warrior + Yr27^a^ | 16/5 | *Yr1, Yr2, Yr3, Yr4, Yr6, Yr7, Yr9, Yr17, Yr25, (Yr27), Yr32, (YrSd), (YrSu), YrSp, YrAvc.S, YrAmb* | *Yr5, Yr8, Yr10, Yr15, Yr24* |

Virulence/ avirulence data provided by K. Flath (JKI Kleinmachnow); tested for pathogenicity on a differential set consisting of 21 resistance genes: *Yr1, Yr2, Yr3, Yr4, Yr5, Yr6, Yr7, Yr8, Yr9, Yr10, Yr15, Yr17, Yr24 Yr25, Yr27, Yr32, YrSd, YrSu, YrSp, YrAvc.S, YrAmb*; isolated in Germany.
