# Supplementary material for: Quantitative Trait Loci Mapping of Adult Plant and Seedling Resistance to Stripe Rust (Puccinia striiformis Westend.) in a Multiparent Advanced Generation Intercross Wheat Population
Source: Front Plant Sci. 2021 Dec 23;12:684671. doi: 10.3389/fpls.2021.684671 (PMC8733622; doi:10.3389/fpls.2021.684671)
Supplement: Supplementary Table 3 — Comparison of the physical positions of the QTL identified in the present study (bold) with those reported previously. [file Table_3.docx]

**Supplementary Table 3** Comparison of physical positions of the QTL identified in the present study (bold) with those reported previously.

| QTL | Marker interval | Physical position [Mb] | Genetic material | References |
| --- | --- | --- | --- | --- |
| *QYr.jki‑1A.1* | IAAV3919 – Tdurum_contig42479_3800 | 1.3 – 12.5 | BMW population (RIL) |  |
| *QYrMa.wgp‑1AS* | IWB57448 – IWB54411 | 7.3 – 9.1 | Avocet‑S × Madsen (RIL) | Liu et al. (2018) |
| *QYr.jki‑1A.2* | Ra_c110766_321 – AX‑94788925 | 540.3 – 593.3 | BMW population (RIL) |  |
| *QRYr1A.1* | Xgwm497 | 551.2 | Avocet × Pastor (RIL) | Rosewarne et al. (2012) |
| *QYr.caas‑1AL* | Xbarc213 – Xwmc59 | 572.2 – 575.4 | Shanghai 3/Catbird × Naxos (RIL) | Ren et al. (2012) |
| *QYr.wsu-1A.2* | IWA3215 | 593.3 | NSGC winter wheat association panel | Bulli et al. (2016) |
| *QRYr1A.1* | wPt-6005 | na | ICARDA spring wheat association panel | Jighly et al. (2015) |
| *QYr.jki‑1D* | IACX11290 – AX‑94893744 | 33.5 – 366.2 | BMW population (RIL) | *Yr25*? |
| *QYr.sun-1D* | Xwmc147 | 0.28 | CPI133872 × Janz (DH) | Zwart et al. (2010) |
| *QYrst.orr-1DS* | Xbcd1434 | na | Stephens × Platte (RIL) | Vazquez et al. (2012) |
| *QYrdr.wgp-1DS* | IWA2268 | 8.5 | Druchamp × Michigan Amber (RIL) | Hou et al. (2015) |
| *QYr.wpg-1D.1* | IWA6960, IWA1396 | 8.2 | PNW winter wheat association panel | Naruoka et al. (2015) |
| *QYr.ucw-1D* | IWA980 | 36.2 | Spring wheat association panel | Maccaferri et al. (2015) |
| *QYr.caas‑1DS* | Xgwm353 – Xgdm33b | na | Shanghai 3/Catbird × Naxos (RIL) | Ren et al. (2012) |
| *QYr.jki‑2A.1* | RFL_Contig174_406 – wsnp_Ex_c11950_19164191 | 3.1 – 4.2 | BMW population (RIL) |  |
| *QYrMa.wgp‑2AS* | IWB35714 | 2.7 | Avocet‑S × Madsen (RIL) | Liu et al. (2018) |
| *Yr17* | Xwmc382 | 2.3 |  | Bariana and McIntosh (1993) |
| *QYr.jki‑2A.2* | AX‑94910560 – AX‑94594842 | 5.7 – 36.1 | BMW population (RIL) |  |
| *QYr.uga‑2AS* | Xbarc124 – Xgwm359 | 3.8 – 28.2 | Pioneer 26R61 × AGS 2000 (RIL) | Hao et al. (2011) |
| *QYr.ucw‑2AS* | wPt‑5839 – Xwmc177 | na – 33.7 | UC1110 × PI610750 (RIL) | Lowe et al. (2011) |
| *QYr.jki‑2B.1* | RAC875_c22429_249 – Tdurum_contig91519_224 | na – 407.2 | BMW population (RIL) |  |
| *QYrid.ui‑2B.1* | wPt‑9668 – Xgwm429 | na – 73.6 | Rio Blanco × IDO444 (RIL) | Chen et al. (2011) |
| *QYr.sgi‑2B.1* | Xgwm148 | 100.8 | Kariega × Avocet-S (RIL) | Ramburan et al. (2004) |
| *QYrlu.cau‑2BS1* | Xwmc154 – Xgwm148 | 36.4 – 100.8 | Aquileja × Luke (F_1_, F_2_, F_3_) | Guo et al. (2008) |
| *QYr.jki‑2B.2* | Excalibur_c40056_59 – BS00034245_51 | 110.9 – 216.5 | BMW population (RIL) |  |
| *QYr.sgi‑2B.1* | Xgwm429 – Xbarc9 | 73.6 – na | Rio Blanco × IDO444 (RIL) | Chen et al. (2011) |
| *QYrlu.cau‑2BS2* | Xgwm148 – Xbarc167 | 100.8 – 448.7 | Aquileja × Luke (F_1_, F_2_, F_3_) | Guo et al. (2008) |
| *Yrlo.wgp‑2BS* | Xwmc474 – Xbarc230 | 172.7 – 218.5 | Louise × Penawawa (RIL) | Carter et al. (2009) |
| *QYr.jki‑2B.3* | AX‑94693685 – AX‑94415212 | 519.0 – 726.5 | BMW population (RIL) |  |
| *QYR1* | Xgwm501 – Xgwm47 | 672.1 – 685.8 | Camp Remy ×Michigan Amber | Boukhatem et al. (2002) |
| *QYraq.cau‑2BL* | Xwmc175 – Xwmc332 | 670.6 –739.4 | Aquileja × Luke (F_1_, F_2_, F_3_) | Guo et al. (2008) |
| *QYr.inra‑2BL* | Xbarc101 – Xwmc175 | 621.5 – 670.6 | Camp Remy × Recital (RIL) | Mallard et al. (2005) |
| *QTL(mix)2B* | Xwmc499 – Xwmc317 | 594.4 – 784.3 | Kris ×·Deben (DH) | Christiansen et al. (2006) |
| *QYr.jki‑2D* | Ku_c19185_1569 – BS00086534_51 | 461.3 – na | BMW population (RIL) |  |
| *QYr.caas‑2DL* | Xgwm539 – Xcfd44 | 513.1 – 608.6 | Shanghai 3/Catbird × Naxos (RIL) | Ren et al. (2012) |
| *QPst.jic‑2D* | Xgwm539 – Xgwm349 | 513.1 – 629.6 | Guardian × Avocet (F_2_, F_3_) | Melichar et al. (2008) |
| *QTL‑2DL* | Xgwm349 | 629.6 | Fukuho‑komugi × Oligoculm (DH) | Suenaga et al. (2003) |
| *QYr.jki‑3B* | Excalibur_rep_c106935_390 – AX‑95187585 | 581.3 – 665.3 | BMW population (RIL) |  |
| *QYrex.wgp‑3BL* | Xgwm299 – Xgwm340 | 804.8 – 826.2 | Express × Avocet-S (RIL) | Lin and Chen (2009) |
| *QYrid.ui‑3B.2* | Xgwm299 | 804.8 | Rio Blanco × IDO444 (RIL) | Chen et al. (2011) |
| *QYr.jki‑3D* | AX.95240079 – BS00093857_51 | 19.8 – 22.0 | BMW population (RIL) |  |
| *QYr.inra‑3DS* | Xbarc125 – Xgwm456a | 174.8 – 282.5 | Renan × Récital. | Dedryver et al. (2009) |
| *QYr.cim‑3D* | Xgdm8 – Xgdm128 | 357.1 – 309.9 | Avocet‑S x Chapio (RIL) | Yang et al. (2013) |
| *QYr.jki‑6A* | wsnp_Ex_c15708_24056750 – Kukri_c24296_273 | na – 612.1 | BMW population (RIL) |  |
| *QYr.cimmyt‑6A* | Xgwm427, Xwmc256, Xgwm617 | 606.6. 549.5. 604.3 | Avocet-S × Pavon76 (RIL) | William et al. (2006) |
| *QYrpl.orr‑6AL* | Xgwm617 – Xcdo836, | 604.3 – na | Stephens × Platte (RIL) | Vazquez et al. (2012) |
| *QTL‑6AL* | Xwmc256 – Xgwm617 | 549.5 – 604.3 | Avocet‑S × Saar (RIL) | Lillemo et al. (2008) |
| *QYr.jki‑7D* | BS00022449_51 – AX‑94883448 | 5.4 – 29.4 | BMW population (RIL) |  |
| *QYr.cim‑7DS* | Xgwm295 – XcsLV34 | 53.6 – na | Avocet‑S × Chapio (RIL) | Yang et al. (2013) |
